# Genomic epidemiology and genetic characteristics of clinical *Campylobacter* species cocirculating in West Bengal, India, 2019, using whole genome analysis

**DOI:** 10.1128/aac.01108-24

**Published:** 2024-12-04

**Authors:** Daichi Morita, Asish Kumar Mukhopadhyay, Goutam Chowdhury, Fumito Maruyama, Miyuki Kanda, Yuki Yamamoto, Hidetoshi Tahara, Piyali Mukherjee, Mainak Bardhan, Takanori Kumagai, Kei Kitahara, Shin-Ichi Miyoshi, Shanta Dutta, Teruo Kuroda

**Affiliations:** 1 Department of Microbiology, Graduate School of Biomedical and Health Sciences, Hiroshima University 592244 , Hiroshima, Japan; 2 Division of Bacteriology, ICMR - National Institute of Cholera and Enteric Diseases 30170 , Kolkata, India; 3 Collaborative Research Centre of Okayama University for Infectious Diseases at ICMR-NICED 30170 , Kolkata, India; 4 Section of Microbial Genomics and Ecology, The IDEC Institute, Hiroshima University, Hiroshima, Japan; 5 Department of Cellular and Molecular Biology, Graduate School of Biomedical and Health Sciences, Hiroshima University 164140 , Hiroshima, Japan; 6 Graduate School of Medicine, Dentistry and Pharmaceutical Sciences, Okayama University 12997 , Okayama, Japan; Universita degli studi di roma La Sapienza, Rome, Italy

**Keywords:** *Campylobacter jejuni/coli*, whole genome sequencing, phylogenetic analysis, plasmid structure

## Abstract

*Campylobacter* species are the most common pathogens responsible for foodborne gastroenteritis worldwide. India is a region with frequent diarrheal infections and a high level of *Campylobacter* infection incidence, but the detailed genomic information is limited. This study aimed to characterize 112 isolates of *Campylobacter* from diarrhea patients at two hospitals in Kolkata, West Bengal, by whole genome analysis. The *Campylobacter* isolates consisted of 90 *C*. *jejuni*, 20 *C*. *coli*, and 2 *C*. *lari* isolates. Multilocus sequence typing analysis revealed that the largest sequence type (ST) populations were ST-2131 in *C. jejuni* and ST-830 in *C. coli* and seven novel STs were found in *C. jejuni* and one in *C. coli*. Notably, ST-2131, which is rarely seen elsewhere, was positive for a sialylated LOS-related gene (*wlaN +neuA + cstIII*) associated with Guillain-Barré syndrome. Antibiotic resistance factors predicted from the genome sequence included *blaOXA* variants (58.9%), *tet(O)* (54.5%), *tet(W)* (0.9%), *ant(6)-Ia* (0.9%), mutation in GyrA (T86I, T86I+D90N, T86I+P104S, T86I+D90*N*+P104S) (79.5%), and mutation in 23S rRNA (A2075G) (12.5%). In addition to the high drug resistance of *Campylobacter* in Kolkata, *Campylobacter* pathogens were circulating that may be associated with Guillain-Barré syndrome. This study indicates the importance of genomic analysis in the surveillance of pathogens, which provides genomic information on genetic diversity, virulence mechanisms, and determinants of antimicrobial resistance.

## INTRODUCTION

Thermophilic *Campylobacter* are widely isolated from the intestinal tracts of various wild and domestic birds and mammals and are known to be the major causative pathogens of foodborne gastroenteritis worldwide. The World Health Organization (WHO) estimates that *Campylobacter* spp. caused 166 million cases of illnesses and 37,604 deaths worldwide in 2010 (1).

*Campylobacter jejuni* and *C. coli* are the most frequent causes of acute diarrheal diseases in humans, with *C. jejuni* accounting for 80%–90% and *C. coli* accounting for 10%–20% of the cases (2). Despite the importance of *C. jejuni* as a foodborne pathogen, little is known about the mechanism of campylobacteriosis. A well-known virulence factor of *C. jejuni* is cytolethal distending toxin (*cdt*). The *cdt* operon of *C. jejuni* is composed of *cdtA*, *cdtB*, and *cdtC* and encodes a multi-subunit holotoxin, and the presence of an intact *cdt* operon results in the release of a functional cytotoxin (3). Although biological functions of type VI secretion system (T6SS) and its effectors in *C. jejuni* are not well known, T6SS has been reported to be associated with host colony formation, cell adhesion and invasion, survival in bile salts, and contact-dependent lysis of red blood cells (4). Acquisition of T6SS in *Campylobacter* occurs by CJPI-1, a genomic island that incorporates T6SS within *Campylobacter jejuni* Integrated Element 3 (CJIE3) and plasmids (5). In addition to diarrheal diseases, important clinical features of *Campylobacter* infections are associated with immunoreactive complications such as acute immune-mediated neuropathy, Guillain-Barré syndrome (GBS), and Miller Fisher syndrome (MFS) (6). In *C. jejuni*, sialylation modification of lipooligosaccharides (LOS) results in molecular mimicry of the glycan component of human GM1 gangliosides in peripheral nerves associated with GBS and MFS (6, 7). GBS and MFS are due to cross-reactivity between antibodies produced in response to LOS and human gangliosides.

Because campylobacteriosis is self-limiting, treatment with antimicrobial agents is not routinely recommended, but fluoroquinolones (FQ) and macrolides are often prescribed for patients with persistent infections, immunodeficiency, and complications. Tetracyclines and aminoglycosides are also used as alternative agents (8). Recently, the emergence and spread of antibiotic-resistant *Campylobacter*, particularly to FQ, has become a cause for concern. In 2017, the WHO listed FQ-resistant *Campylobacter* as one of the high-priority pathogens for research and development of new antibiotics.

Multilocus sequence typing (MLST) is an important index for phylogeny and epidemiology of *Campylobacter*. MLST analysis of *Campylobacter* has shown that several clonal complexes (CC), such as CC21, CC353, and CC45, are known to be epidemic lineages, confirming their ubiquity and global distribution (9). However, MLST analysis does not provide medically important information such as virulence and antibiotic resistance determinants. *Campylobacter* spp. are genetically variable pathogens, with frequent horizontal gene exchange and recombination, which means that strains with the same sequence type (ST) could have different virulence patterns. Whole genome sequencing (WGS) is considered the most informative and discriminatory analysis for bacterial pathogens, allowing comprehensive phylogenetic analysis of numerous traits related to virulence and antibiotic resistance.

In India, official bacterial food poisoning statistics have not been conducted, and the actual incidence of food poisoning and resistance related to *Campylobacter* is unclear. *Campylobacter* food poisoning is expected to be one of the major causes of diarrheal infections, especially considering the sanitation problems and use of poultry as a major food source. Kolkata is the state capital of West Bengal, one of the most populous states, and is also a major poultry-producing state in India (https://dahd.nic.in/document/annual_report). The genomic information on *Campylobacter* isolated from humans in India has not been well characterized. This study aimed to determine the genetic diversity, resistance, and virulence determinants of *Campylobacter* in Kolkata, a region with frequent diarrheal infections, by WGS. This study provides an important baseline for the surveillance of the genetic diversity and characteristics of *Campylobacter* in India and may be useful for epidemiological studies and clinical decision-making.

## MATERIALS AND METHODS

### Bacterial isolates

Strains were isolated from fecal samples from patients treated at the Infectious Diseases and Beleghata General Hospital and Dr. B. C. Roy Post-graduate Institute of Pediatric Sciences in Kolkata, India in 2019. Fecal samples were streaked on brain–heart infusion agar with 0.2% activated charcoal, 5% horse serum, and antimicrobial drugs (cycloheximide, bacitracin, cephazolin sodium, novobiocin, colistin sulfate) and incubated under microaerophilic conditions (5% O_2_, 10% CO_2_, and 85% N_2_) at 37°C for 48 hours. Characteristic *Campylobacter* colonies were confirmed by Gram stain, oxidase testing followed by standard biochemical testing, and species-specific PCR was performed to identify five species from the *Campylobacter* genus (10).

### DNA extraction and genome sequencing

DNA extraction procedures were performed using GenElute bacterial genomic DNA kit (Sigma). The DNA quality and concentration were measured using Qubit fluorometer. Short-read libraries were prepared based on the NEB Next Ultra II FS DNA Library Prep with Sample Purification Beads (NEB) and sequenced using Illumina NextSeq 1000/2000 P2 Reagent v3 (300 cycles). Long-read libraries were prepared based on the Rapid Barcoding Kit 96 (Nanopore) and sequenced using Oxford Nanopore MinION.

### *De novo* genome assembly, annotation, pangenome, and phylogenetic analyses

Paired-end 150 bp FASTQ files were passed using Bactopia 1.7.1 to assess data quality and assemble contigs (11). In hybrid assembly, the long reads were base-called using Guppy 6.1.5 and filtered using NanoFilt v2.8.0 (-l 3000 --headcrop 75) (12). For assembly, Flye v2.9 was used (13), and the assembled sequences were polished using Illumina reads with Pilon v1.24 by default parameters (14). The assembled sequences were determined to be circular according to Flye (Table S1). Assembled genomes were annotated using DFAST (https://dfast.ddbj.nig.ac.jp).

Minimum spanning trees were generated and visualized in GrapeTree (15). To identify the genus core genome, we used Panaroo v1.2.9 to generate a gene presence-absence matrix using the default settings and the -a core flag to generate a core gene alignment (16). A core gene phylogeny was constructed from the core gene alignment using IQ-Tree software 2.0.3 (-m HKY -bb 1000 -alrt 1000 -wbt -wbtl -alninfo) (17). Phylogenetic tree visualization was conducted using the Interactive Tree of Life v6.5 (iTOL) (https://itol.embl.de).

### *In silico* identification of antimicrobial resistance genes, virulence genes, and MLST

The assembled genomes were then passed through a bioinformatic pipeline using BLAST techniques to identify antimicrobial resistance (AMR) genes, AMR-associated point mutations, virulence genes, and MLST according to ABRicate (https://github.com/tseemann/abricate) and STARAMR 0.7.2 (https://github.com/phac-nml/staramr). ABRicate databases were based on ResFinder and vfdb databases (last updated on 3 February 2022). STARAMR databases were based on Pointfinder v050218 (database date of 7 January 2022) and pubMLST databases (database date of 3 July 2023) (18–21).

### *In silico* identification of Penner genotype and T6SS

The presence of the specific capsular polysaccharide synthesis (CPS) sequences for a particular serotype was determined by performing a local stand-alone BLAST analysis using a database encompassing the nucleotide sequences of CPS genotyping multiplex PCR amplification region (22). Primers and their respective PCR product sizes are listed in Table S5. To identify the 13 components of type VI secretion system (T6SS), BLASTN was used with default parameters for the T6SS gene from *C. jejuni* subsp. *jejuni* strain 14980A (CP017029.1). The T6SS gene was considered positive when a minimum of 80% identity and coverage was observed.

### Statistical analysis

All statistical analyses and figures were performed in R (version 4.1.2). The analyzed data set includes RefSeq genomic data of 1944 *C. jejuni* and 1012 *C*. *coli* registered at NCBI until 2021.

To compare the prevalence of individual antibiotic resistance between this study and the NCBI data, chi-squared tests were performed. In all cases, a *P* value of <0.05 was considered statistically significant.

## RESULTS

### Genomic characteristics of *Campylobacter* in Kolkata

The genomes of 112 isolates of *Campylobacter* collected in 2019 from diarrhea patients at two hospitals in Kolkata, West Bengal, were subjected to short-read sequencing on all isolates and long-read sequencing on 60 isolates (Table S1). For consistency, genome analysis was conducted on *de novo* assembly genome sequences based on short-read sequencing, unless noted otherwise.

**Fig 1:**
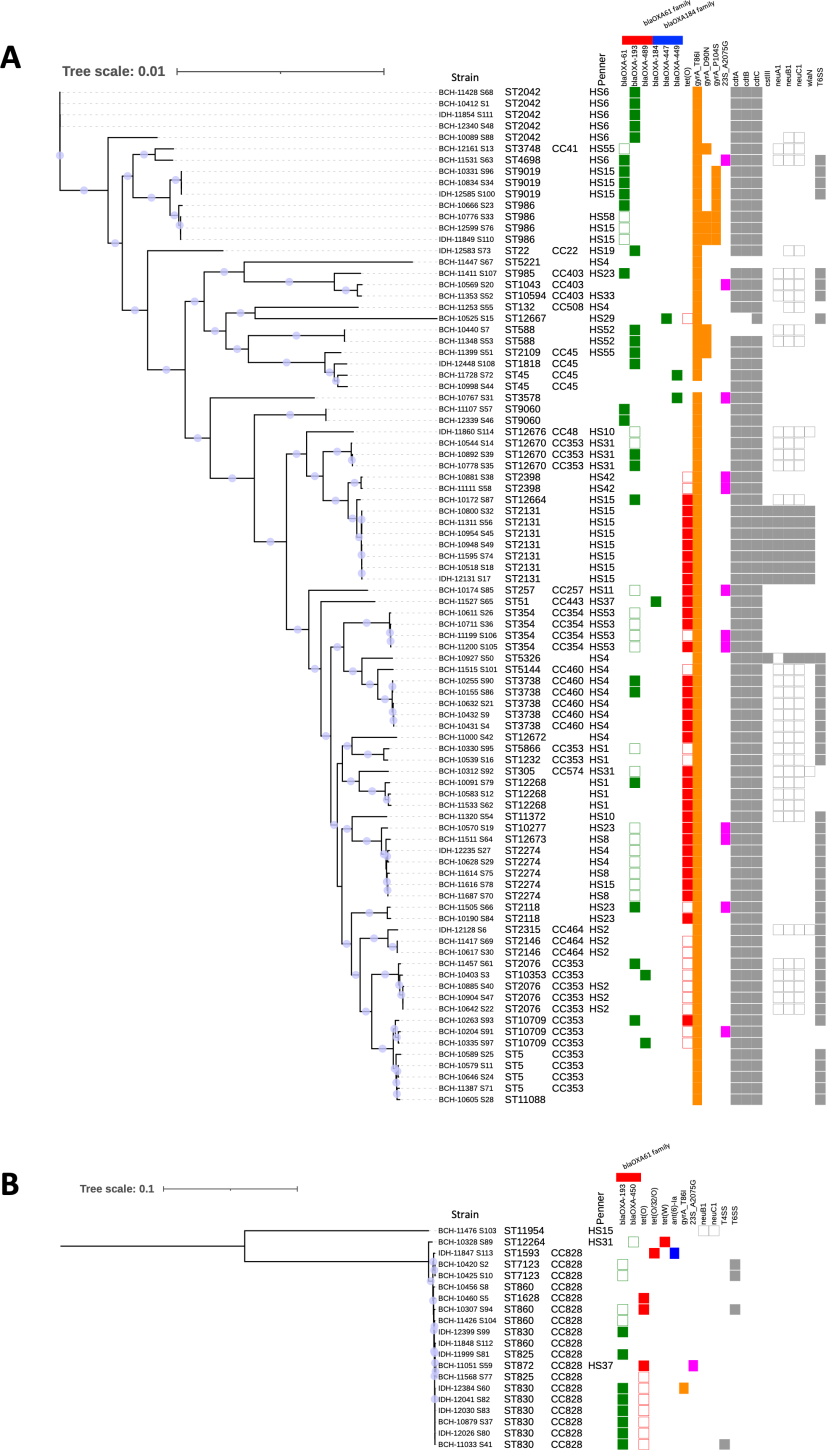
Core genome phylogenetic tree of *C. jejuni* (**A**) and *C. coli* (**B**). The phylogenomic analysis is based on core genome comparison using Panaroo. The maximum likelihood tree was generated by IQ-TREE. Bootstrap support values were calculated from 1,000 replicates, and only values above 95% are shown as circles. Right fields indicate sequence type, clonal complex, *in silico* Penner typing, resistance genes, and important virulence genes. In the *blaOXA61*-like family, filled boxes indicate the presence of promoter mutations, and blank boxes indicate no promoter mutations; in *tet(O*) and *tet(W*), filled boxes indicate acquisition in the genome, and blank boxes indicate acquisition in the plasmid; in *cstIII*, *neuABC*, and *wlaN*, filled boxes indicates the presence of a gene (80% or more sequence identity), and a blank box indicates the presence of a gene with low identity (less than 80% sequence identity). A comprehensive list of all virulence genes identified in each strain is given in Table S3.

**Fig 2:**
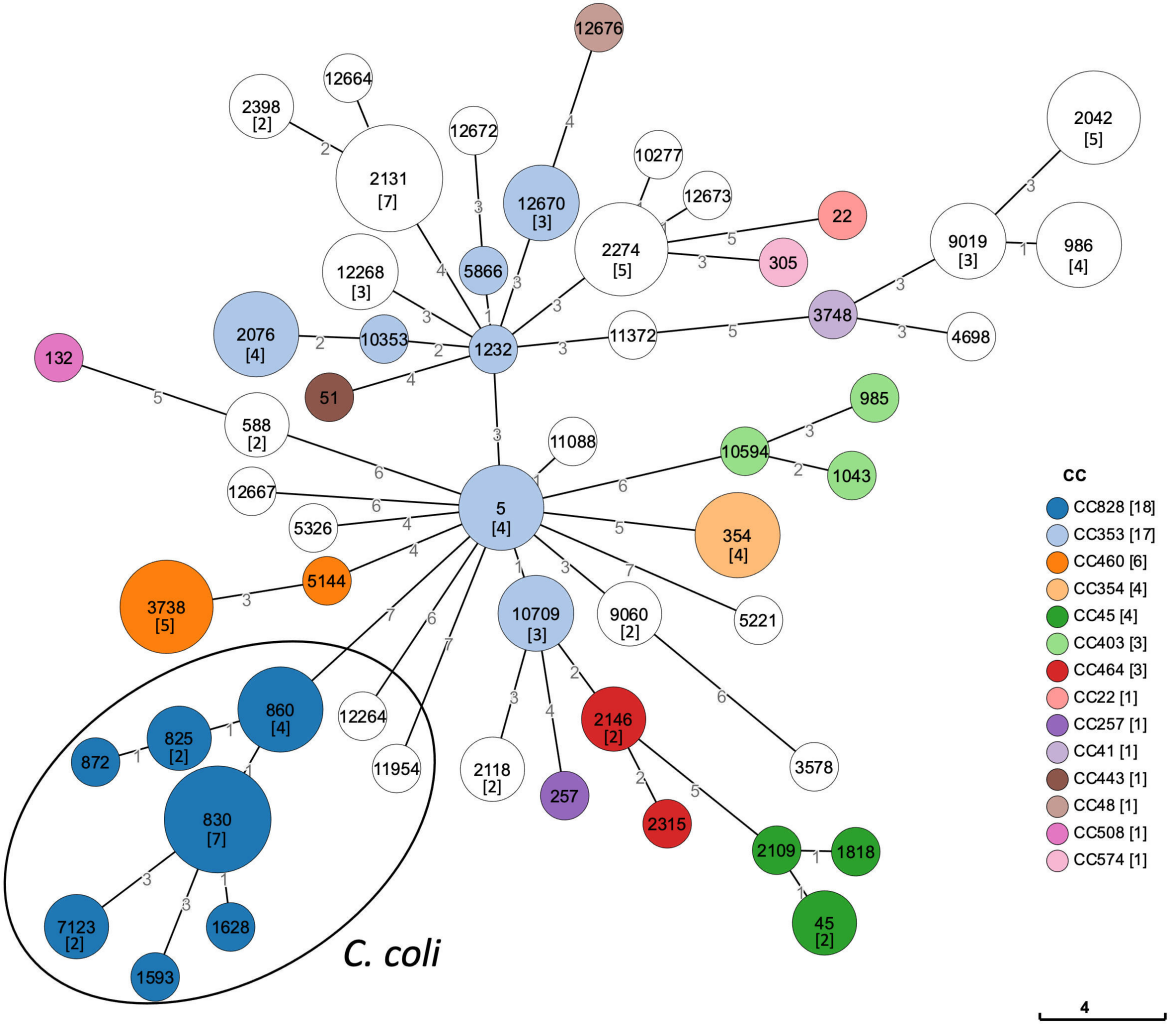
Minimum spanning tree of *C. jejuni* and *C. coli*. The nodes indicate the detected sequence types and are proportional to the number of strains. For nodes with more than two strains, the number of strains is indicated in [ ]. Clonal complexes are colored with the corresponding node indicated in the legend.

The assembled genomes were identified as *C. jejuni* (*n* = 90), *C. coli* (*n* = 20), and *C. lari* (*n* = 2) by taxonomy checks using DFAST, and a maximum likelihood phylogenetic tree based on the core genome was generated for *C. jejuni* and *C. coli* (Fig. 1). Results of MLST showed that isolates of *C. jejuni* belonged to 39 known STs and 7 novel STs, isolates of *C. coli* belonged to 8 known STs and 1 novel ST, and isolate of *C. lari* belonged to 1 novel ST (Fig. 2; Table S2). The most frequent ST in *C. jejuni* and *C. coli* were ST-2131 (7/90) and ST-830 (7/20), respectively. In *C. jejuni*, the strains were classified into 12 CCs, with the largest CC in *C. jejuni* being the CC353 (17/90), followed by the CC460 (6/90) and the CC354 (4/90). All but two *C. coli* isolates belonged to CC828.

The results of *in silico* Penner typing showed that 75 isolates (*C. jejuni* 72/90, *C. coli* 3/20) were assigned to 19 Penner types, and 37 isolates were not assigned to a specific Penner type. The predominant Penner types were HS15 (13.6%) and HS4 (10.9%) (Fig. 1; Table S2).

### Distribution of resistance determinants

WGS analysis revealed 11 resistance genes including four variants of *blaOXA-61*; three variants of *blaOXA-184*, *tet(O*), *tet(W*), *tet(O/32/O*), and *ant (6)-Ia*; and five resistance-related point mutations in GyrA and 23S rRNA (Table 1; Fig. 1; Table S2).

**TABLE 1:** Distribution of antibiotic resistance genes of *Campylobacter* isolated from clinical patients

	*C. jejuni* (*n* = 90)	*C. coli* (*n* = 20)	*C. lari* (*n* = 2)	Total (*n* = 112)
Aminoglycoside resistance				
*ant(6)-Ia*	0	1	0	1
β-lactam resistance				
*blaOXA-61*-like family				
*blaOXA-61*	12	0	0	12
*blaOXA-193*	35	12	0	47
*blaOXA-450*	0	1	0	1
*blaOXA-489*	2	0	0	2
*blaOXA-184*-like family				
*blaOXA-184*	1	0	0	1
*blaOXA-447*	1	0	0	1
*blaOXA-449*	2	0	0	2
Tetracycline resistance				
*tet(O*)	50	10	0	60
*tet(O/32/O*)	0	1	0	1
*tet(W*)	0	1	0	1
Macrolide resistance				
23S rRNA (A2075G)	12	2	0	14
Quinolone resistance				
GyrA (T86I)	77	1	0	78
GyrA (T86I, D90N)	4	0	0	4
GyrA (T86I, P104S)	4	0	0	4
GyrA (T86I, D90N, P104S)	3	0	0	3

**Fig 3:**
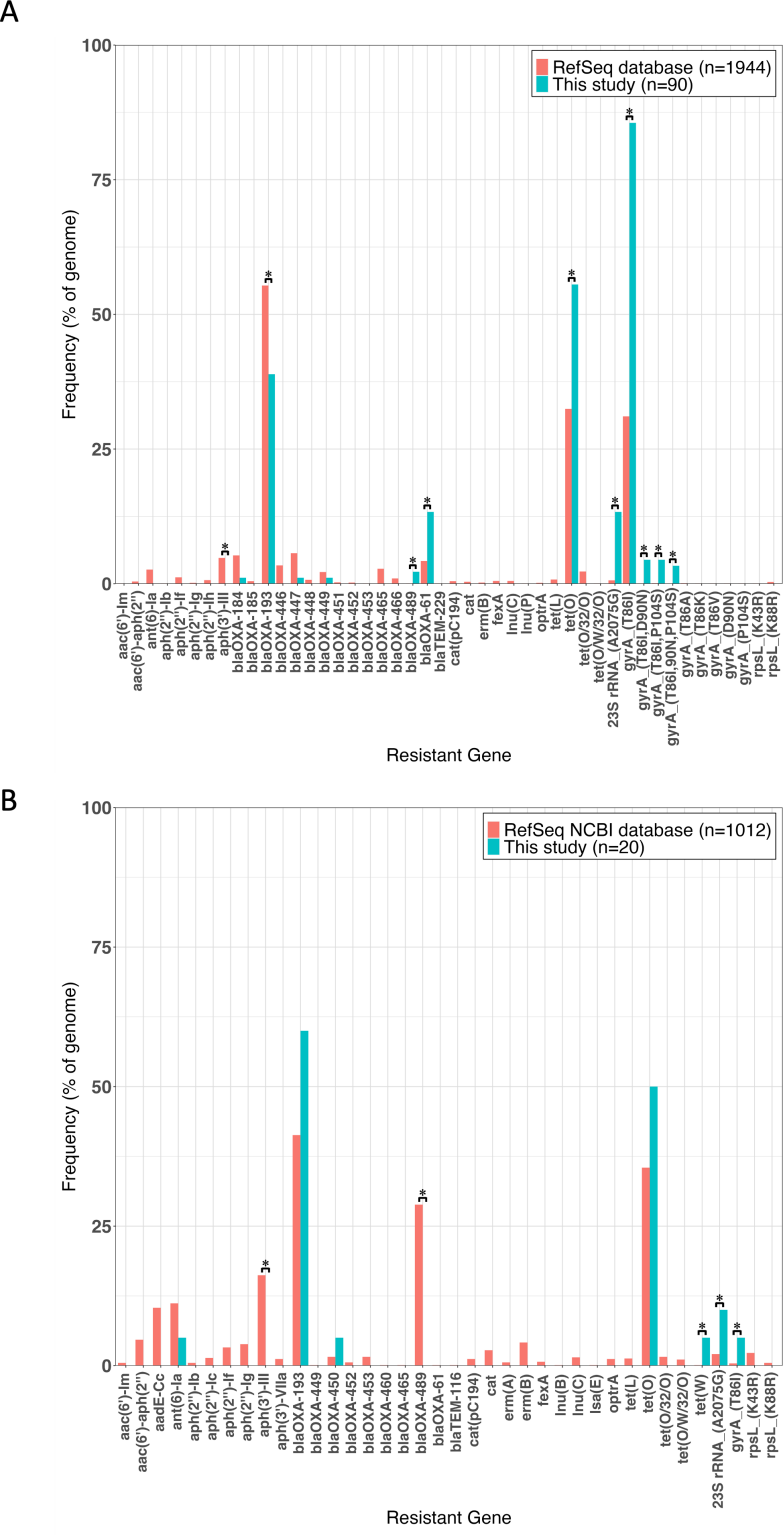
Comparison of resistance gene prevalence between *C. jejuni* (**A**) and *C. coli* (**B**). The percentages of genes detected in the RefSeq genome are represented by blue bars, and the percentages of genes detected in this study are represented by red bars. ^*^P < 0.05 according to the chi-squared test.

Resistance gene prevalence in *C. jejuni* and *C. coli* in this study was compared with the RefSeq genomes (Fig. 3). In *C. jejuni*, *aph(3″)-III* and *blaOXA-193* were significantly low, whereas *balOXA-61*, *blaOXA-489*, *tet(O*), 23S rRNA mutation, and GyrA mutation were significantly high. In *C. coli*, *aph(3″)-III* and *blaOXA-489* were significantly low, and *tet(W*), 23S rRNA mutation, and GyrA mutation were significantly higher.

Sixty-six isolates, accounting for 58.9% of the total, harbored *blaOXA* genes (*C. jejuni* 53/90, *C. coli* 13/20, *C. lari* 0/2). It is reported that the *blaOXA-61*-like family of *Campylobacter* is not normally expressed and is upregulated by G→T transversion of the promoter region (57 bp upstream from the annotated start codon) and is resistant to antibiotics such as ampicillin (23). Of the 62 isolates with *blaOXA-61*-like family genes, 37 isolates had this mutation (*blaOXA-61: C. jejuni n* = 8/12, *blaOXA-193: C. jejuni n* = 19/35, *C. coli n* = 8/12, *blaOXA-450: C. coli n* = 0/1, and *blaOXA-489: C. jejuni n* = 2/2).

Sixty-two isolates, accounting for 56.3% of the total, harbored tetracycline resistance ribosomal protection protein genes (54.5% with *tet(O*) and 0.9% with *tet(O/32/O*) and *tet(W*)). Acquisition of *tet(O*) was caused by pTet family plasmids (*n* = 22) and genomic insertions (*n* = 39). Genomic insertions were identified at five regions, except in two isolates, BCH-10583 and BCH-11533, whose insertion sites were not determined. The site corresponding to *Cj0730-0731* in *C. jejuni* NTCT11168 (AL111168.1) was the hot spot for *tet(O*) insertion (Fig. S1). In *Cj0493-0494*, large prophage-like insertions including integrase and in *Cj1523-1529*, insertions of sequences derived from the pTet family plasmid were observed, whereas *Cj730-731*, *Cj1062-1066*, and *Cj1620-1622* contained small insertions consisting of several genes, including *tet(O*). The insertion of *tet(W*) occurred in *Cj1620-1622*. Detection of *tet(W*) in *Campylobacter* is rare, and this is the first time that the insertion sequence of *tet(W*) has been determined.

The aminoglycoside resistance gene, *ant (6)-Ia*, was only detected in one isolate in *C. coli*. The gene was inserted into the genome along with *tet(O/32/O*) and was located between *Cj1624-1627*. The *tnpV* in this sequence may be responsible for the insertion.

Point mutations in the quinolone resistance-determining region of the *gyrA* associated with FQ resistance were detected in 89 isolates, accounting for 79.5% of the total, mostly in *C. jejuni*. Almost all mutations were in GyrA (T86I), and multiple mutations were rare. A point mutation within the peptidyl transferase region in V domain of the 23S rRNA, A2075G associated with macrolide resistance was detected in 14 isolates, accounting for 12.5% of the total, 13% in *C. jejuni* and 10% in *C. coli*.

### Distribution of virulence determinants

Virulence genes of *Campylobacter* were searched in the vfdb database using ABRicate. Several genes of *C. coli*, *C. lari*, and some *C. jejuni* were not detected with gene identity above 80%, the default parameter in ABRicate, but were detected above 70%. A total of 128 virulence genes were detected: 121 genes in *C. jejuni*, 107 genes in *C. coli*, and 62 genes in *C. lari* (Table S3; Fig. 3). Of the genes detected, 71 genes in *C. jejuni*, 72 genes in *C. coli*, and 58 genes in *C. lari* were found in all of the strains, mainly cell surface lipooligosaccharides (LOS), CPS, and flagellar genes.

Among the cytolethal distending genes, toxin, LOS sialylation-related genes, and type IV secretion system (T4SS), which are highly related to the pathogenicity of *Campylobacter*, are discussed as follows. A well-known virulence factor of *C. jejuni* is cytolethal distending toxin (*cdt*). The *cdt* genes in 90 *C*. *jejuni* isolates were present in high frequencies (*cdtA n* = 88/90, *cdtB n* = 88/90, *cdtC n* = 89/90). The genes involved in sialylated LOS have been reported as *wlaN* encoding 1,3-galactosyltransferase, *neuA* encoding acylneuraminic acid cytidylyltransferase, and *cstIII* encoding sialyltransferase. In this study, seven isolates of *wlaN + neuABC + cstIII* and one isolate of *wlaN + neuBC + cstIII* were detected from *C. jejuni* in more than 99% identity with the vfdb sequence. Although it is unclear whether LOS sialylation is involved, *neuABC* and *wlaN* with less than 80% identity were identified in some strains (*neuABC + wlaN*: 3 strains, *neuABC*: 29 strains, *neuBC*: 3 strains). T4SS is a highly diverse secretion system present in both Gram-negative and Gram-positive bacteria, but the relationship between T4SS and pathogenicity in *Campylobacter* remains unclear. T4SS genes that could be associated with pathogenicity, *cjp54*, *virB4*, *virB8*, *virB9*, *virB10*, *virB11*, and *virD4*, were only detected in one isolate of *C. coli*.

### Prevalence and structures of the T6SS

By matching 13 genes of T6SS components (*tagH*, *tssA-tssM*), 2 isolates were found to be completely T6SS positive in *C. jejuni*, 39 isolates were T6SS positive lacking *tssI*, 1 isolate was T6SS positive lacking *tssI* and *tssJ*, and 5 isolates had partial components (*tagH*, 1 strain; *tagH + tssI*, 4 strains). Additionally, one isolate of *C. coli* was positive for T6SS lacking *tssI*, and two isolates were found to have a partial component (*tssEFG + tssI*), but none of them were detected in *C. lari* (Table S4). The component gene of T6SS, *tssI*, also had homology to CJIE3 (CP000025.1) component genes, *cje1141* (first half of *tssI*) and *cje1142* (second half of *tssI*), and the detection rate of *tssI* was low because short-read-based genome assembly produced incomplete gaps in these gene sequences. Of the 60 isolates with hybrid assembly, 27 were positive for T6SS (complete T6SS in 16 isolates, T6SS with truncated *tssI* in 10 isolates, and *tssEFG + tssI* in 1 isolate). Of these, 5 isolates had T6SS on plasmids, and 22 isolates had T6SS on genomes. All of the isolates detected in the genome constituted CJPI-1. These CJPI-1 islands consisted of a large number of genes other than T6SS and CJIE3, forming a diverse array of variations. Only *C. coli* BCH-10420 with a partial component (*tssEFG + tssI*) showed a large internal arrangement of CJPI-1 by inversion (Fig. S2).

### Plasmid variations and structures

There are several families of plasmids that are specific to *Campylobacter* (24). Analysis of plasmids in the 60 isolates with hybrid assembly revealed 25 plasmids, including 15 pTet family plasmids related to tetracycline resistance, 5 pCC plasmids commonly found in *C. coli*, 4 T6SS-related plasmids, and 1 *C*. *lari* plasmid. The structures of the plasmids in each family were highly conserved with known plasmids (Fig. S3).

In the pTet family, *tet(O*) and T4SS, which are involved in conjugative transmission, were identified in all plasmids. The structure of the T6SS-associated plasmid consists of a portion of CJPI-1, and since many plasmids with similar structures are found in the NCBI database, this island is presumed to be transferable to both genomic and plasmid types. In addition, one plasmid in pTet family plasmid contained T6SS. The integration of T6SS into the pTet family plasmid has often been reported. There were five pCC family plasmid-like plasmids detected, three in *C. coli* and two in *C. jejuni*. The latter plasmid was named pCJ. The *C. lari* plasmid (pCL) has only a few identical sequences in the NCBI database, which are from *C. lari* and *C. peloridis*.

## DISCUSSION

Genomic surveillance of pathogens enables clinical decision-making in the management of infectious diseases by clarifying the status of risk factors such as prevalent strains, antibiotic resistance, and virulence factors in local populations and allowing for comparative analysis of pathogens on local and global scales. The whole genome analysis and comprehensive genetic analysis of *Campylobacter* in this study could provide insights into the actual status of *Campylobacter* food poisoning in India. Herein, we analyzed the genomic diversity of 112 isolates of *Campylobacter* obtained from diarrhea patients at two hospitals in Kolkata, West Bengal, in 2019.

It has been reported that the global prevalence lineages consist of several CCs, such as CC21, CC353, and CC45, in *C. jejuni* (9). In *C. coli*, two CCs, CC828 and CC1150, account for the majority of isolates, and CC828 accounts for 70% of isolates (2). In this study, CC353 was the largest CC for *C. jejuni* in Kolkata. The *C. coli* isolates mostly comprised CC828. Note that CC21 was not present. In *C. jejuni*, the largest ST was ST-2131, which is not classified in a specific CC and not reported in other countries. In addition, seven novel STs in *C. jejuni* isolates, one novel ST in *C. coli* isolates, and one novel ST in *C. lari* isolates were present, indicating that the endemic population in this region has genetic diversity that differs from the global trend. This difference from the global trend of *C. jejuni* may indicate the possibility of an India-specific endemic strain of *Campylobacter* and the existence of diverse *Campylobacter* contamination processes in the region due to poultry feeding at the individual level and the slaughter and sale of poultry in street markets. Nonetheless, we recognize the limitations of our study. Genomic information on *Campylobacter* in India was limited and could not be compared among multiple regions. Therefore, a more diverse regional analysis is required to determine whether this study reflects *Campylobacter* in India overall.

Lior and Penner serotyping methods have been used for *Campylobacter* (25, 26), and recently, multiplex PCR Penner method, which provides more stable results, has been proposed (22). To date, serotyping information on *Campylobacter* in India has been limited, indicating the need for a more simple and accurate assessment such as the multiplex PCR Penner assay. Furthermore, some *Campylobacter* were untypeable by *in silico* Penner method, and there is a possibility of novel Penner serotypes, requiring a more detailed analysis of the CPS region, the determinant of Penner serotype.

Although phenotypic antimicrobial susceptibility testing of isolates was not performed in this study, our previous study (27) and other reports (28, 29) have shown a high correspondence between resistant genotypes and resistant phenotypes in *Campylobacter*. Genetic evidence of resistance to fluoroquinolones and tetracyclines in *C. jejuni* and fluoroquinolones in *C. coli* was significantly more frequent than in other regions (9, 27, 30, 31). The 23S rRNA mutation is responsible for high macrolide resistance in *Campylobacter* (28, 29, 32), and the mutation was confirmed in 14 strains (*C. jejuni* 12/90, *C. coli* 2/20). This tended to be higher than in developed countries such as Ireland, Japan, the United States, and the United Kingdom (27, 30, 31). The high frequency of mutations in *C. jejuni* may require more attention considering that *C. jejuni* is the major causative agent of campylobacteriosis. Although the frequency of the *blaOXA-61* in this study was lower than in other reports, single nucleotide mutations in the promoter region of *blaOXA-61* (G→T transversion) frequency was very high at 59.6% (27, 33). It has been shown that the expression of *blaOXA-61* is increased in the presence of this mutation, resulting in higher β-lactam resistance in *C. jejuni* (23, 33). In a previous study, *C. jejuni* with the G→T mutation had higher ampicillin resistance than *C. jejuni* without this mutation (27, 34). This suggests that despite β-lactam not being a therapeutic agent for *Campylobacter*, it may be under some selective pressure in India.

Important clinical symptoms of *Campylobacter* infection include immune-responsive complications such as GBS and MFS (6). Molecular mimicry of the saccharide component of human GM1 gangliosides by sialylated LOS in *C. jejuni* has been reported to be a major factor in triggering these two neuropathies. In Japan, 52%–77% of patients with *C. jejuni*-associated GBS were Penner serotype HS:19 (accounting for approximately 3% of *C. jejuni* enteritis cases) (35). In South Africa, 9 of 17 children (53%) admitted to the intensive care unit with GBS had evidence of *C. jejuni* infection, and all isolates were Penner serotype HS:41 (36). Other *C. jejuni* serotypes identified in association with GBS include HS:1, HS:2, HS:4, HS:4/50, HS:5, HS:10, HS:16, HS:23, HS:37, HS:44, and HS:64 (6). The strains in which sialylated LOS-related genes (7, 37), *wlaN* encoding 1,3-galactosyltransferase, *neuA* encoding acylneuraminic acid cytidylyltransferase, and *cstIII* encoding sialyltransferase, were detected in this study were HS:4 and HS:15, especially HS:15, which was the most frequently isolated ST type, ST-2131. Although there is little known about GBS associated with *C. jejuni* in India, *C. jejuni* infection may be a major risk for GBS.

### Conclusions

The epidemiology of *Campylobacter* in developing countries, the acquisition of drug resistance, and risk factors for human infection remain poorly understood. This study revealed that the prevalent strains of *Campylobacter* in West Bengal differ from those in other countries, considering the numerous genetic lineages and wide genetic diversity, as well as their possession of resistance and virulence genes. Resistance factors were identified against FQ, tetracycline, and macrolide, and the resistance factors against FQ and macrolide were identified at a higher frequency. Except in immunocompromised patients and pregnancy, antimicrobial therapy for *Campylobacter* enteritis remains controversial. However, severe cases are common in undernourished populations in developing countries, and the spread of antibiotic resistance limits effective antimicrobial treatment options. Although the virulence mechanisms of *Campylobacter* are unknown, it is notable that the most common ST-2131, which is rarely reported in other regions, were all Penner serotype HS:15 with sialylation-related genes associated with GBS. Whole genome analysis provides accurate and detailed information about pathogens, and our results demonstrate the utility of pathogen genomics as an important tool for disease surveillance, control, and clinical decision-making for pathogens in India.

## Data Availability

The genome sequences analyzed in this study are available under BioProject accession number PRJDB15932. Accession numbers and BioSample identifiers are listed in Table S1 in the supplemental material. Refseq genomes of *C. jejuni* (*n* = 1,944) and *C. coli* (*n* = 1,012) registered in the NCBI repository before 2021 were used for global antibiotic resistance gene analysis.

## SUPPLEMENTAL MATERIAL

Figures S1 to S3 aac.01108-24-s0001.ppt Comparison of tet(O) insertion sites, T6SS structure, and plasmids.

Tables S1 to S5 aac.01108-24-s0002.xlsx Accession numbers of strains used, blast analysis of resistance and virulence genes, and primer information for in silico PCR.

ASM does not own the copyrights to Supplemental Material that may be linked to, or accessed through, an article. The authors have granted ASM a non-exclusive, world-wide license to publish the Supplemental Material files. Please contact the corresponding author directly for reuse.
