# Supplementary material for: Genomic epidemiology and genetic characteristics of clinical Campylobacter species cocirculating in West Bengal, India, 2019, using whole genome analysis
Source: Antimicrob Agents Chemother. 2024 Dec 4;69(1):e01108-24. doi: 10.1128/aac.01108-24 (PMC11784092; doi:10.1128/aac.01108-24)
Supplement: Figures S1 to S3 — Comparison of tet(O) insertion sites, T6SS structure, and plasmids. [file aac.01108-24-s0001.ppt]

## Slide 1
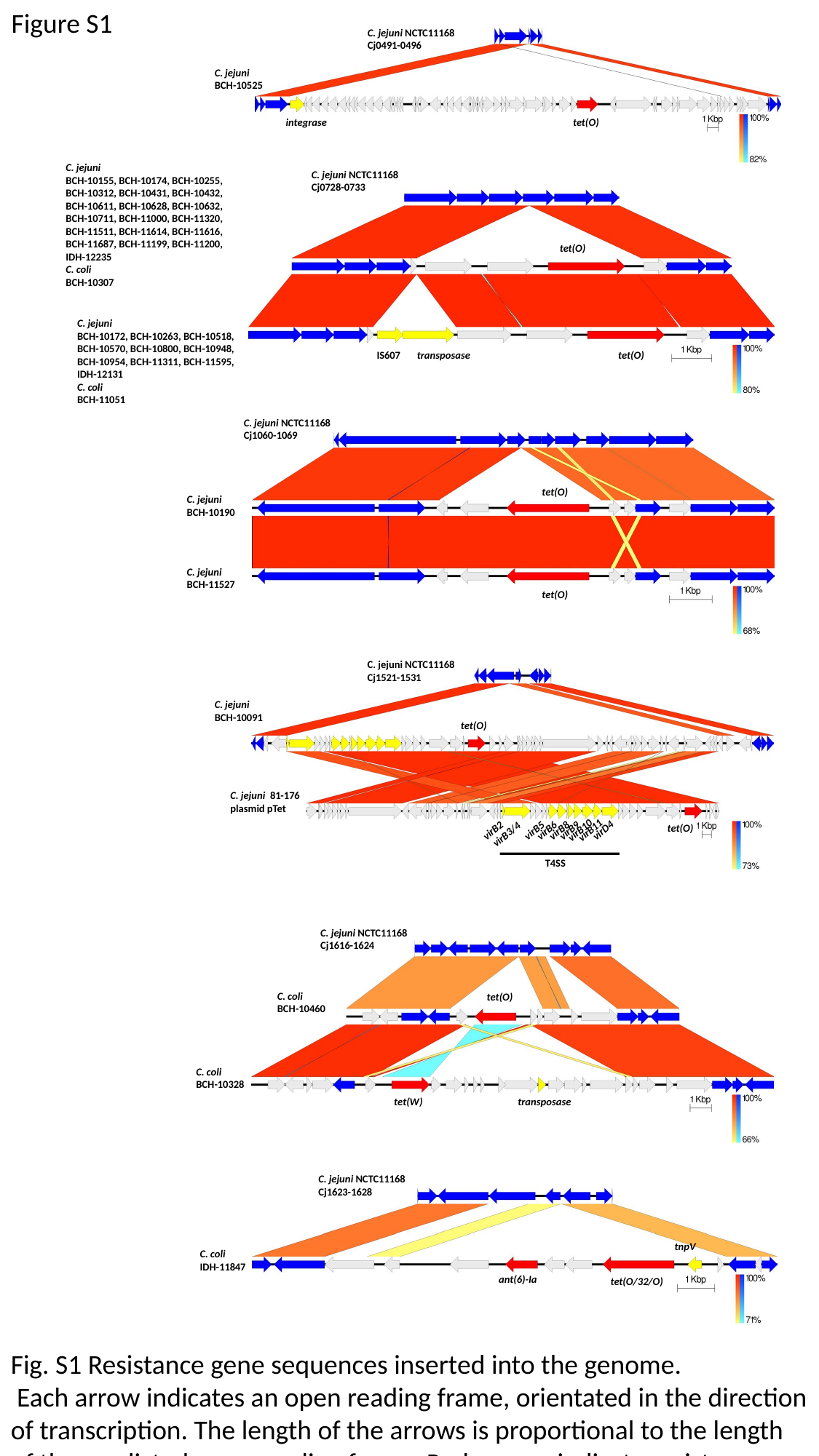

Figure S1
C. jejuni NCTC11168
Cj0491-0496
C. jejuni
BCH-10525
integrase
tet(O)
C. jejuni
BCH-10155, BCH-10174, BCH-10255,
BCH-10312, BCH-10431, BCH-10432,
BCH-10611, BCH-10628, BCH-10632,
BCH-10711, BCH-11000, BCH-11320,
BCH-11511, BCH-11614, BCH-11616,
BCH-11687, BCH-11199, BCH-11200,
IDH-12235
C. coli
BCH-10307
C. jejuni NCTC11168
Cj0728-0733
tet(O)
C. jejuni
BCH-10172, BCH-10263, BCH-10518,
BCH-10570, BCH-10800, BCH-10948,
BCH-10954, BCH-11311, BCH-11595,
IDH-12131
C. coli
BCH-11051
IS607
transposase
tet(O)
C. jejuni NCTC11168
Cj1060-1069
tet(O)
C. jejuni
BCH-10190
C. jejuni
BCH-11527
tet(O)
C. jejuni NCTC11168
Cj1521-1531
C. jejuni
BCH-10091
tet(O)
C. jejuni 81-176
plasmid pTet
tet(O)
virB10
virB11
virB2
virB5
virB6
virB8
virB9
virD4
virB3/4
T4SS
C. jejuni NCTC11168
Cj1616-1624
C. coli
BCH-10460
tet(O)
C. coli
BCH-10328
tet(W)
transposase
C. jejuni NCTC11168
Cj1623-1628
tnpV
C. coli
IDH-11847
ant(6)-Ia
tet(O/32/O)
Fig. S1 Resistance gene sequences inserted into the genome.
 Each arrow indicates an open reading frame, orientated in the direction of transcription. The length of the arrows is proportional to the length of the predicted open reading frame. Red arrows indicate resistance genes, yellow arrows indicate genes associated with gene transfer and blue arrows indicate genes homologous to C. jejuni NCTC11168. The color spectrum between sequences represents identity.

## Slide 2
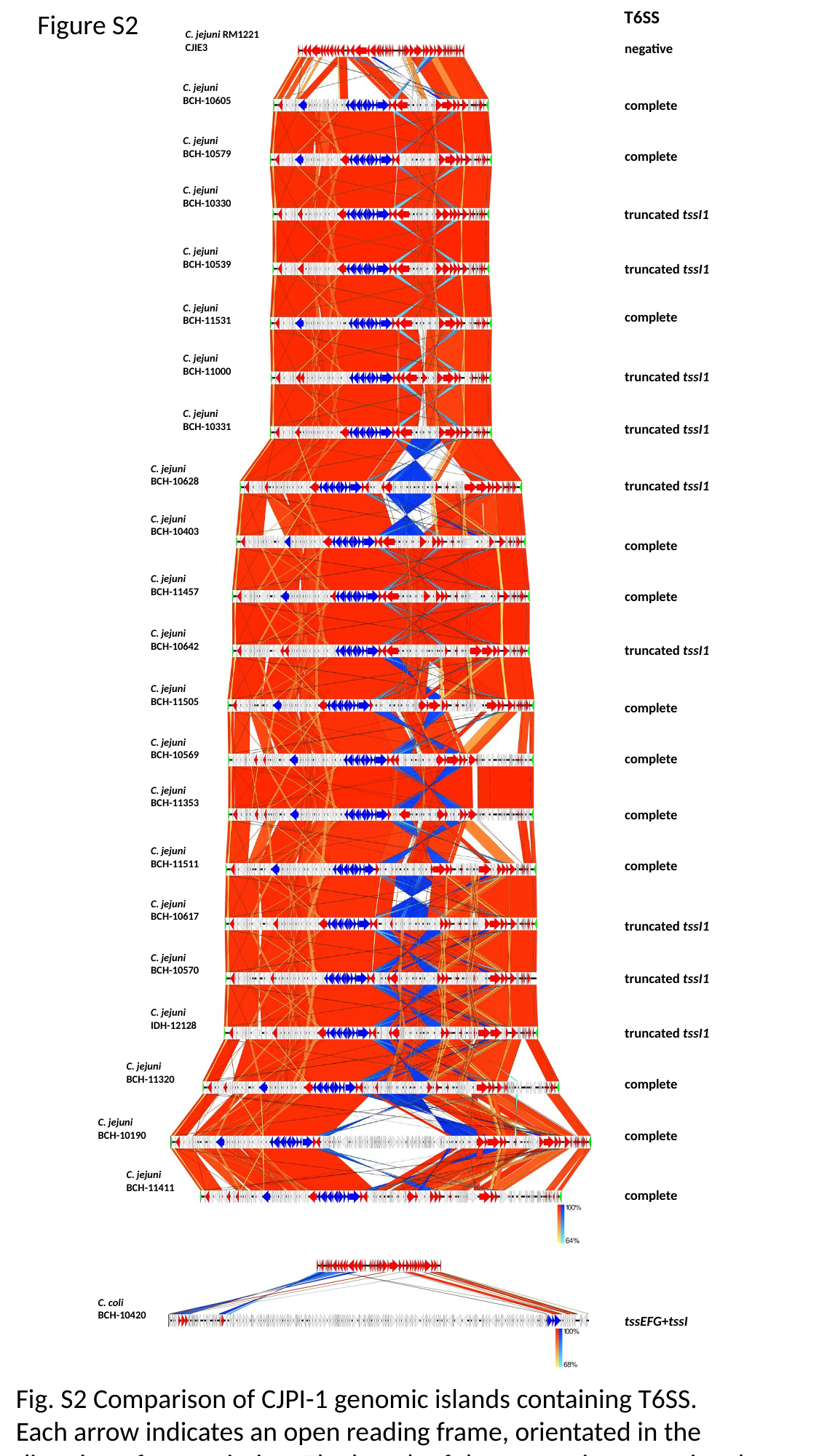

T6SS
Figure S2
C. jejuni RM1221
CJIE3
negative
C. jejuni
BCH-10605
complete
C. jejuni
BCH-10579
complete
C. jejuni
BCH-10330
truncated tssI1
C. jejuni
BCH-10539
truncated tssI1
C. jejuni
BCH-11531
complete
C. jejuni
BCH-11000
truncated tssI1
C. jejuni
BCH-10331
truncated tssI1
C. jejuni
BCH-10628
truncated tssI1
C. jejuni
BCH-10403
complete
C. jejuni
BCH-11457
complete
C. jejuni
BCH-10642
truncated tssI1
C. jejuni
BCH-11505
complete
C. jejuni
BCH-10569
complete
C. jejuni
BCH-11353
complete
C. jejuni
BCH-11511
complete
C. jejuni
BCH-10617
truncated tssI1
C. jejuni
BCH-10570
truncated tssI1
C. jejuni
IDH-12128
truncated tssI1
C. jejuni
BCH-11320
complete
C. jejuni
BCH-10190
complete
C. jejuni
BCH-11411
complete
C. coli
BCH-10420
tssEFG+tssI
Fig. S2 Comparison of CJPI-1 genomic islands containing T6SS.
Each arrow indicates an open reading frame, orientated in the direction of transcription. The length of the arrows is proportional to the the length of the predicted open reading frame. Red arrows indicate　 genes homologous to CJIE3 in C. jejuni RM1221 and blue arrows indicate T6SS component genes. The color spectrum between sequences represents identity.

## Slide 3
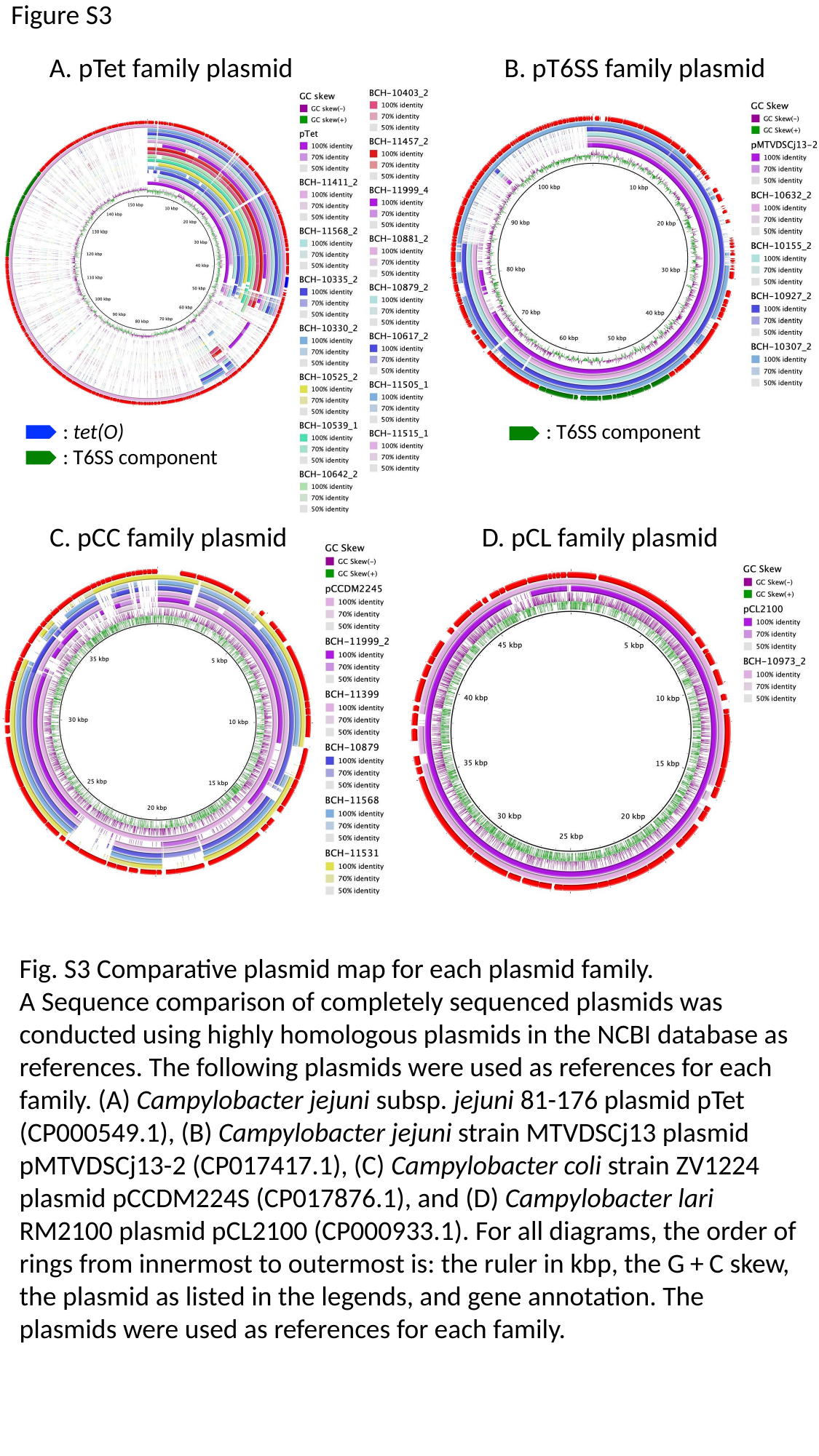

Figure S3
A. pTet family plasmid
B. pT6SS family plasmid
: tet(O)
: T6SS component
: T6SS component
C. pCC family plasmid
D. pCL family plasmid
Fig. S3 Comparative plasmid map for each plasmid family.
A Sequence comparison of completely sequenced plasmids was conducted using highly homologous plasmids in the NCBI database as references. The following plasmids were used as references for each family. (A) Campylobacter jejuni subsp. jejuni 81-176 plasmid pTet (CP000549.1), (B) Campylobacter jejuni strain MTVDSCj13 plasmid pMTVDSCj13-2 (CP017417.1), (C) Campylobacter coli strain ZV1224 plasmid pCCDM224S (CP017876.1), and (D) Campylobacter lari RM2100 plasmid pCL2100 (CP000933.1). For all diagrams, the order of rings from innermost to outermost is: the ruler in kbp, the G + C skew, the plasmid as listed in the legends, and gene annotation. The plasmids were used as references for each family.
